# Consent Processes for Mobile App Mediated Research: Systematic Review

**DOI:** 10.2196/mhealth.7014

**Published:** 2017-08-30

**Authors:** Sarah Moore, Anne-Marie Tassé, Adrian Thorogood, Ingrid Winship, Ma'n Zawati, Megan Doerr

**Affiliations:** ^1^ Sage Bionetworks Seattle, WA United States; ^2^ Public Population Project in Genomics and Society Montreal, QC Canada; ^3^ Center of Genomics and Policy McGill University Montreal, QC Canada; ^4^ Melbourne Health University of Melbourne Melbourne Australia

**Keywords:** mHealth, informed consent, smartphone, cell phone, mobile applications, privacy, research ethics

## Abstract

**Background:**

Since the launch of ResearchKit on the iOS platform in March 2015 and ResearchStack on the Android platform in June 2016, many academic and commercial institutions around the world have adapted these frameworks to develop mobile app-based research studies. These studies cover a wide variety of subject areas including melanoma, cardiomyopathy, and autism. Additionally, these app-based studies target a variety of participant populations, including children and pregnant women.

**Objective:**

The aim of this review was to document the variety of self-administered remote informed consent processes used in app-based research studies available between May and September 2016. Remote consent is defined as any consenting process with zero in-person steps, when a participant is able to join a study without ever seeing a member of the research team. This type of review has not been previously conducted. The research community would benefit from a rigorous interrogation of the types of consent taken as part of the seismic shift to entirely mobile meditated research studies.

**Methods:**

This review examines both the process of information giving and specific content shared, with special attention to data privacy, aggregation, and sharing.

**Results:**

Consistency across some elements of the app-based consent processes was found; for example, informing participants about how data will be curated from the phone. Variations in other elements were identified; for example, where specific information is shared and the level of detail disclosed. Additionally, several novel elements present in eConsent not typically seen in traditional consent for research were highlighted.

**Conclusions:**

This review advocates the importance of participant informedness in a novel and largely unregulated research setting.

## Introduction

Patients want to share their health data to accelerate scientific discovery efforts [1]. This desire to share data has led to projects such as Open Humans [2], whereby people can upload data from many sources to share with researchers, and PatientsLikeMe [3], a popular website to find communities of patients clustered by disease or condition. In both of the aforementioned cases, the Internet serves as a powerful tool for remote connection and a central location for data.

Recent advances in technology, especially mobile platform development, offer further opportunity for data aggregation and sharing by activated patients [4]. Smartphones: portable, loaded with sensors, and virtually ubiquitous have the potential to revolutionize both the way in which individuals monitor their health and the way they share that data with researchers [5].

In March 2015, Apple launched ResearchKit [6] and in June 2016 Android launched ResearchStack [7]. Both open source frameworks can be used to create apps for research on mobile devices. Unsurprisingly, by offering a dynamic, customizable, and responsive platform for engaging participants in research and enabling rapidly scalable, longitudinal investigations, these devices are being heralded as a potential boon to human health researchers [8].

In order to fully capitalize on the promise of mobile technology to enable scalable research, approaches to informed consent must be adapted in parallel [9]. Novel approaches to informed consent must still ensure the core tenets of informedness, comprehension, and voluntariness [10-12]. Further, informed consent must address unique issues that arise from conducting research using mobile platforms, such as data security and transferability. Researchers are faced with a novel challenge of consenting participants in a completely self-administered setting with no required contact with the research team [9].

Research apps present new challenges and opportunities in informed consent [9]. In this review, a granular inventory of the informed consent processes of publically available research apps has been presented to serve as a foundation for a community-wide discussion on how best to uphold the tenets of informed consent in mobile research settings.

## Methods

### Establishing a Task Team

A task team was convened under the Global Alliance for Genomics and Health (GA4GH) [13]. The GA4GH, established in 2013, works to enable responsible and effective sharing of genomic and clinical data to advance understanding of human health. Aware of the developments in mobile device-facilitated human subjects’ research, GA4GH convened an international task team in May 2016 to examine issues of informed consent within this novel setting.

### Inclusion and Exclusion Criteria

First, we sought to establish a listing (inventory) of app-based research studies with entirely self-administered consent processes currently available worldwide (Multimedia Appendix 1). We excluded app-based research studies that had one or more mandatory in-person informed consent steps.

The inventory included research apps using the ResearchKit framework that are publically available on the Apple iOS platform. We do not include any ResearchStack apps (based on Android platform) in the inventory as none were publically available during our period of review.

Within the inventory (Multimedia Appendix 1), there are multiple sheets based on the consent information available for each app. The sheet of the app-based research study informed consent inventory labeled “Apps with no consent info” contains limited information of apps that claim to use the ResearchKit framework. However, these apps do not have a defined consent process, and are therefore excluded from the complete review.

### Identifying Mobile Research Apps

Due to the emergent nature of the field, as there were few scientific publications and no centralized listings or public catalogs of research apps, we were unable to employ traditional review approaches. Instead, we used an automated continuous web search of the terms “ResearchKit” and “ResearchStack” over three months (May-July, 2016). The primary reviewer received email notification each time the search terms yielded a new result. Through this search, we were able to identify newly released apps via press releases, blog postings, and other web mentions (eg, social media). When possible, we corresponded with authors of web content about research apps to ensure we achieved saturation of the field. We created a listing of the names of individual apps and then searched for them in the respective app download site (iOS or Android). Additionally, we relied on contacts both in the US and in other countries aware of this project to inform us of new research apps. The list of research apps was continuously updated to include newly released study apps throughout the development of the inventory.

### Identification and Refinement of Domains

**Figure 1:**
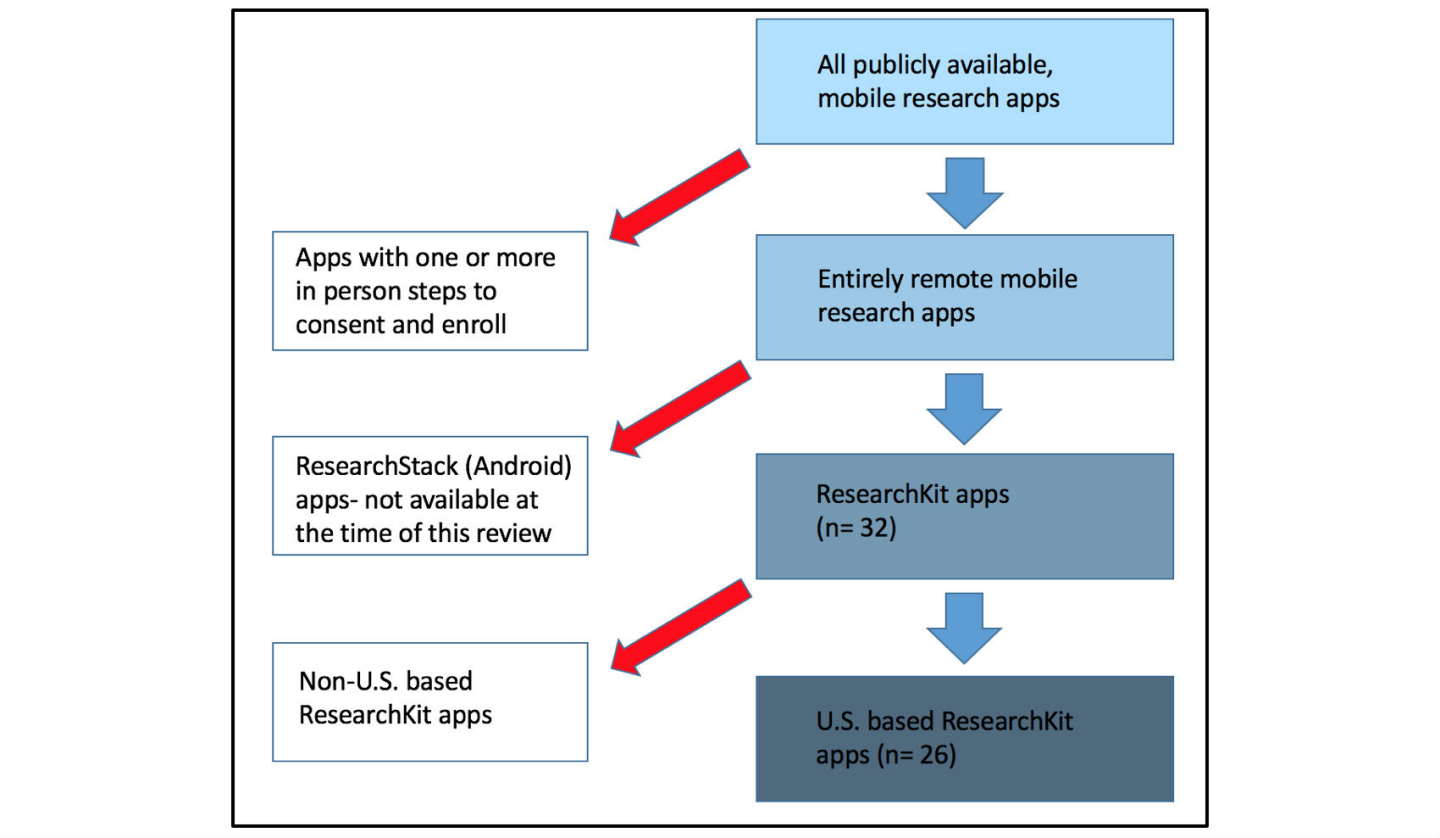
Summary of methods.

We identified 3 components within app-mediated research where information is presented to a potential participant: the eConsent, long form consent (LFC), and privacy policy (PP). Across the apps surveyed, after meeting the eligibility criteria, prospective participants typically self-guide through the eConsent. The eConsent is a series of screens within the research app that a participant navigates prior to enrollment. It contains information traditionally disclosed during an informed consent process such as information about the study, study procedures, alternatives to participation, and risk and benefits. The LFC document is commonly interpreted to be required by US regulation. Participants are presented with the document on the phone prior to signing and joining the study. Participants receive a copy of the document following electronic signature and enrollment (Figure 1). Additionally, we found privacy policies, traditionally used within apps and by websites that alerted users on how data is gathered, used, managed, and potentially disclosed by the organization hosting the app or website, as a repository of study information critical to the informed consent process. Participants can view the PP from the app download site before downloading the app and, for some of the apps, review from within the app as well.

We began by surveying each app’s informed consent materials for the 8 required elements of consent under the US Common Rule (description, risks, benefits, alternatives, confidentiality, compensation, contact information, and voluntary participation/withdrawal). We considered these domains to be predefined by regulatory requirements. Due to the nature of app-based research, we expanded our assessment of some core consent topics to include subtopics critical to app-based research. For example, “risks” needed to include not only general discomforts one may experience as being part of the study, but also address the risk to privacy. “Confidentiality” was expanded to include data collection, handling, and protection of participant privacy. We considered these domains to be emergent in reviewing the apps. Further, we chose to include domains based on thematic consistency. For example, if we saw that the majority of apps surveyed addressed a certain topic, we chose to include this topic in our review.

In reviewing eConsent processes and LFC documents for both predefined and emergent domains within “confidentiality,” we concluded that we must expand our survey to include the PP as another possible source of this information in order to accurately assess disclosure. We expanded the inventory to include the source of information as well as its presence or absence.

The task team iteratively reviewed and discussed findings to refine domains included in the inventory via monthly video meetings as well as email discussions between meetings. Each of the 6 authors of this paper engaged in the discussions. Domains were finalized through group consensus based on a combination of information presented in the consent processes as well as calling attention to domains that have been omitted from the consent processes. After deciding on the domains, we determined how we would categorize the information gleaned from the informed consent materials to populate each domain (ie, Boolean, multi-option, or qualitative description).

### Data Extraction and Coding

Due to mobile device country of registration app access restrictions, the primary reviewer downloaded and performed the initial assessment of only US-based apps. Non–US-based apps were reviewed by a member of the task team residing in the country of origin of the app. We used key word text searches and subsequent coding to identify data within the consent materials for each domain. For example, to determine whether a particular app disclosed information about encryption, we searched the text of the consent document and/or PP for the word “encrypt.” If the word was found within the text, the domain would be filled with “YES” to indicate that the app disclosed information about data encryption. When needed, a second reviewer cross examined the original and extracted content.

## Results

We surveyed a total of 34 app-based informed consent processes for apps available between May and August, 2016, of which 26 underwent complete review (see Multimedia Appendix 1). Some apps were closed during the period of review which limited the information we were able to obtain (3/26 apps closed; GlucoSuccess, Share the Journey and mTech).

Within the inventory, the sheet, “US-based apps with partial/complete consent,” gives an overview of the apps inventoried, including the app name (column A), the version we reviewed (B), and the date of review (C). The study’s focus is described in Subject/Disease Area (D), Targeted Population (E), Identifiable/Personal Data Collected (F), and Data Collection (G). Given that one of the key components of the informed consent is the ability for potential participants to ask questions and request clarification, we identify whether columns for the Sponsor, Principal Investigator, and Contact Information (H-J) are listed, as well as for the Institutional Review Board (IRB) for the study, if any (K). However, one of the key criteria for the apps included in this review is that participants are not required to have contact with anyone on the study team prior to consent and enrolling in the study.

“Elements of Consent LFC” catalogues US Common Rule consent requirements as presented in the LFC with the exception of confidentiality, which is addressed in its own sheet due to the number of subcriteria identified. Under the Common Rule (45 CFR part 46 subpart A) the description of the research should include a statement indicating that the study is intended as research, the purpose of the study, the expected duration, and the procedures (column B). Additional Common Rule requirements are covered in “Risks” (C), “Benefits” (D), “Alternatives to Participation” (E), “Compensation” (F), and “Whom to Contact” (G). The voluntary nature of research (H) and any data retention after withdrawal (I) are described. It is important to note that all of the apps we surveyed were for observational studies, and none administered any treatment. For each domain, a checkmark indicates that all required subcriteria are met; missing subcriteria are listed by number. The sheet labeled “Elements of Consent eConsent” addresses whether or not these same US Common Rule consent requirements are addressed within the eConsent.

The theme of confidentiality is of critical importance in app-based research. We assessed 24 domains on the “Confidentiality” sheet. The first 4 columns address data collection. “Data Collection: Active/Passive” (column B) describes participant effort in data generation. Active tasks require deliberate action on the part of the participant, for example, by responding to surveys or doing a sensor-based task like a tapping test. Passive tasks are those in which the participant donates data without conscious effort, such as through the tracking and transmitting of GPS data. Some studies request permission access to data from other apps on the phone, for example HealthKit (C), or to phone features (D) like the camera and the microphone. Column E addresses whether the research app integrates any other data provided by an outside source. At present, this domain includes the integration of genomic data from testing companies or research sponsor organizations.

Columns labeled “Data Security” address the transmission of data from the participant phone to the backend collection including the app developer (F), whether or not the data will be encrypted (G), the name of the backend collector (H), and whether or not the data collected will be coded or pseudonymized (I). It is important to note that the information described in the table represents what is disclosed to participants. In reviewing the encryption of data, we found variation in which point in the process data is encrypted. Some apps appear to encrypt participant data on the participant phone and maintain this encryption during transmission, while others appear only to encrypt the data when it arrived to the backend server. The domain labeled “Backend Collection” is particularly important to note. In this column, we noted when a researcher has contracted out to a third party to provide data collection services (H).

We include 4 columns describing disclosure. “Required Disclosure” includes informing participants of possible sharing of data with US federal agencies, such as the US Department of Health and Human Services, and the institutional review board (IRB) or other ethics committee (J). “Commercial resale of data” addresses whether the participant is informed about the possibility that the data they have donated for research may be sold to third parties for advertising or other commercial endeavors (K). Additionally, we noted whether this information was found in the LFC or the PP. “Open data sharing for scientific discovery” (L) describes whether the research app has any kind of data sharing outside the primary investigator. If so, we have described what kind of data sharing the participant may consent to: with researchers within the institution of the primary research, with researchers at other institutions, or with qualified researchers worldwide including cross-border data transfer.

The remaining columns on the “Confidentiality” sheet address the option of using of the app without enrolling in research (M), if the recontact of participants is addressed (N), the return of the participant’s own data (O), and incidental findings (P). “Data Preservation” addresses how long the data is authorized to be used by the researcher (Q). About half of the apps surveyed addressed this information either by giving a date that the data will expire or by giving a time period (eg, “6 years after the close of the study”). “Closure” addresses if information is given to participants about what will happen to their data after the period of authorization is over, or in the case of the close of databank or research institution (R). None of the apps reviewed address this domain.

**Figure 2:**
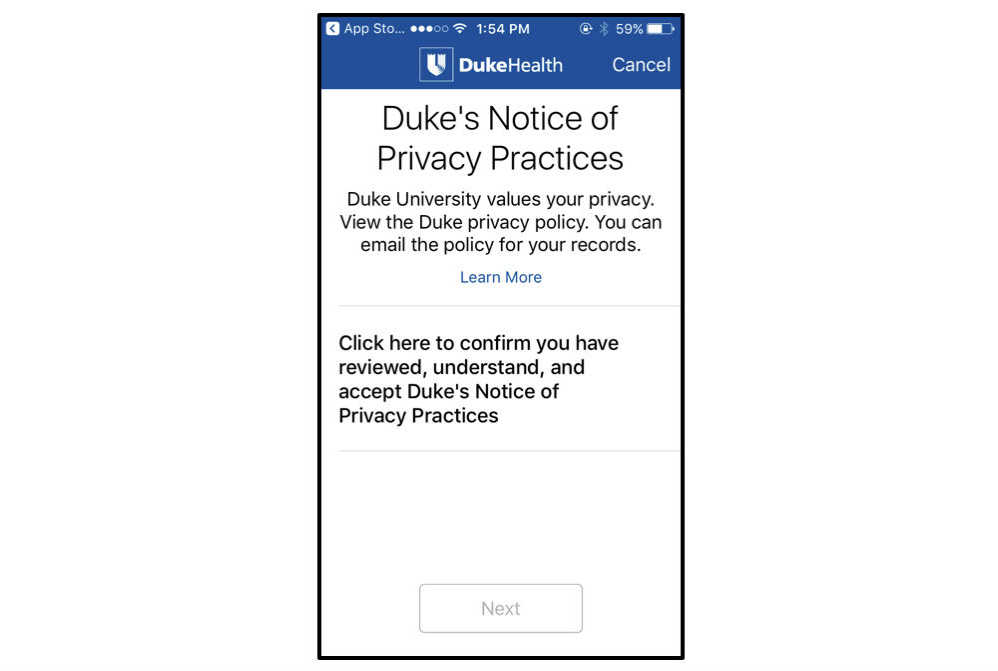
6th Vital Sign, Duke University privacy policy embedded in consent process.

**Figure 3:**
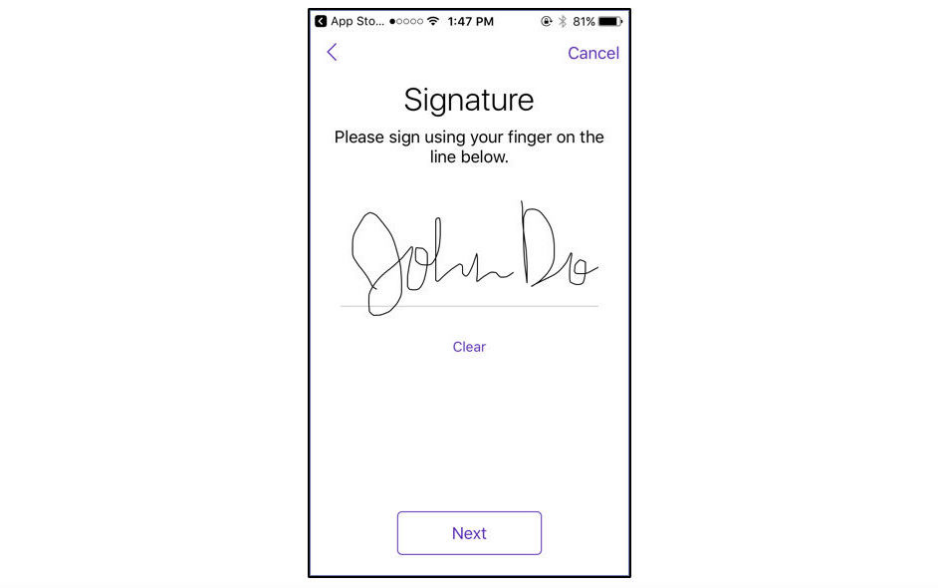
Participants use finger signature to authorize consent to research (image from mPower, Sage Bionetworks).

The “Privacy Policy” sheet inventories the availability and content addressed within each app’s PP. Column B addresses whether the PP in the iOS app store, prior to download, was reviewed. As it is an Apple requirement for any app collecting any user or usage data, including personal health information, all domains are marked “YES.” “Privacy Policy presented as part of consent interaction” addresses whether the prospective participant is made aware of the PP before consenting to participate (C) (eg, Figure 2). In many cases, the PP addresses what personal identifiers the app collects (D) and states that it cannot fully guarantee privacy (E). Column F for “notes” includes whether the PP is tailored specifically to the app and whether it includes the date on which it was last updated. The “Unique to eConsent” sheet addresses elements novel to self-administered, entirely remote consent, including the use of an assessment to assess informedness (D) and approach to consent documentation. If an app uses a quiz at the end of the eConsent, we have indicated whether it is mandatory (E), how many questions are part of the assessment (F), and the information the questions are assessing (G). As eConsent may not meet US informed consent requirements, participants also have access to a LFC (H). We indicate whether finger signature is required (I) (Figure 3, mPower, Sage Bionetworks) and whether the participant would receive the signed LFC via email automatically (J).

The sheet, “Non–US-based apps,” contains the same descriptive domains (eg, app name, version, and subject matter) as the overview “US-based apps” sheet. Due to the geolocation requirements of app download, we struggled to review these apps completely. We were successful in completing a partial review of 2 non–US-based apps: ACL Rupture and Depressed, both from Germany. In the US based review, there was tremendous diversity in the detail and information provided by the apps about the nature of the research being conducted and elements of informed consent.

## Discussion

### Limitations to Methods

Due to the limitations of the approach, the listing may not be comprehensive. Any omissions are due to inability to find these apps through the methods described above rather than deliberate exclusion. Additionally, because of the duration of this project, 3 apps that were available the beginning of the study are no longer available (GlucoSuccess, MGH; mTech, University of the Pacific; Share the Journey, Sage Bionetworks). The inventory is current as of September 1, 2016.

Using mobile platforms for research creates new opportunities for both the types and volume of data collected within a highly scalable, delocalized framework. To harness the full potential of these platforms, informed consent must be similarly scalable without betraying the core principles of informedness, voluntariness, and comprehension [12].

**Figure 4:**
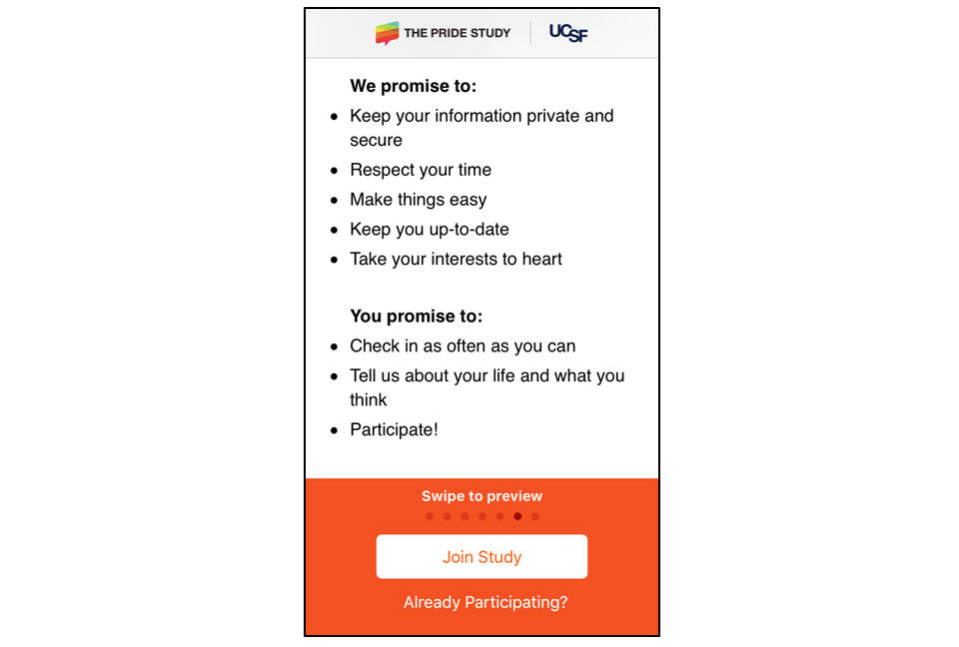
The Pride Study, UCSF researcher and participant agreement.

In this novel research ecosystem, without a face-to-face interaction, participants must rely solely on eConsent, LFC, and PP documents to understand what researchers intend to do with their data and how they will protect it. Because of this, it is vital that researchers take an intentional approach to participant informedness, as seen in The Pride Study app (Figure 4).

In current practice, the eConsent, LFC, and PP are used in tandem with one another to inform the prospective participant, with key information potentially found in any of these locations. For example, the disclosure that researchers “cannot fully guarantee privacy,” was found in either in the PP or the LFC, without a consistent standard across the apps surveyed. Additionally, it was found that in most apps, participants were only required to review the eConsent, but not the LFC and/or PP (See sheet “Privacy Policy,” Column C) prior to participating.

### Privacy, Security, and Data Use

Privacy is the ability to control the recording and sharing of personal information with others. This requires knowledge of what will be recorded, how it will be used and for how long, who will have access to this information, and what risks are of discovery and misuse by third parties [14].

The opportunity for researchers to gather more and different types of data through app-mediated research than they would be able to gather in a traditional clinical study poses a unique risk to privacy. For example, in mPower, a study on Parkinson Disease, researchers are also able to gather GPS data.

Because of the diversity and volume of data being collected, participants are potentially more easily identifiable. It may be impossible to deidentify an individual’s mobile phone data, the standard way of protecting personal privacy in research [14]. Further, a number of the apps surveyed use a backend data collection service other than the primary research sponsor (See sheet “Confidentiality,” Column K). Third party cloud and hosting services provide an economical solution for hosting and storing large amounts of collected data. In addition to an outside group having access to the data collected, the use of a third party poses the threat via hacking of information sent over the Internet [15]. Furthermore, data collected through app-mediated research is collected in the context of a particular disease or condition, such as Parkinson Disease, Melanoma, Autism, or Cardiomyopathy, potentially compromising a participant’s confidentiality through reidentification.

**Figure 5:**
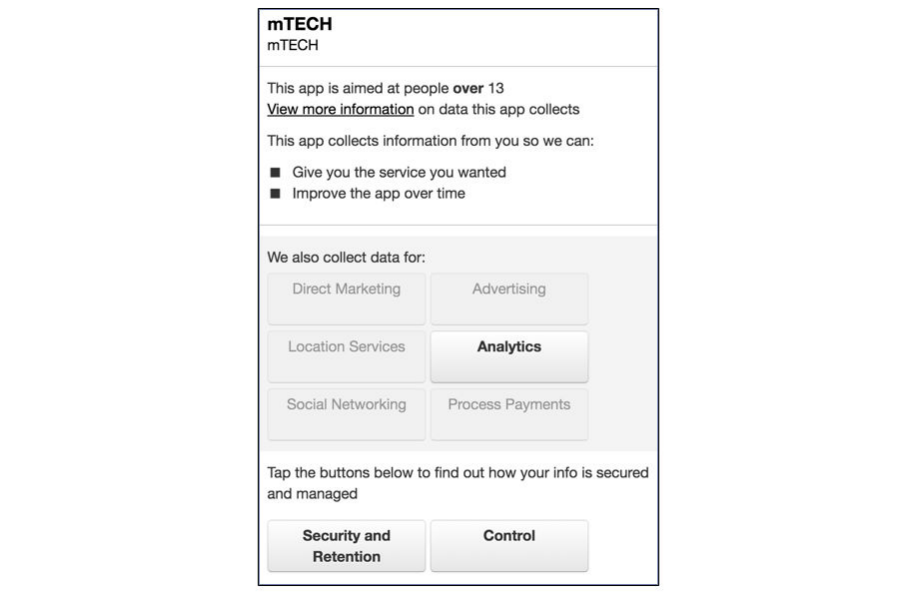
Privacy policies: mTech, University of the Pacific and Team Study, Harvard University.

**Figure 6:**
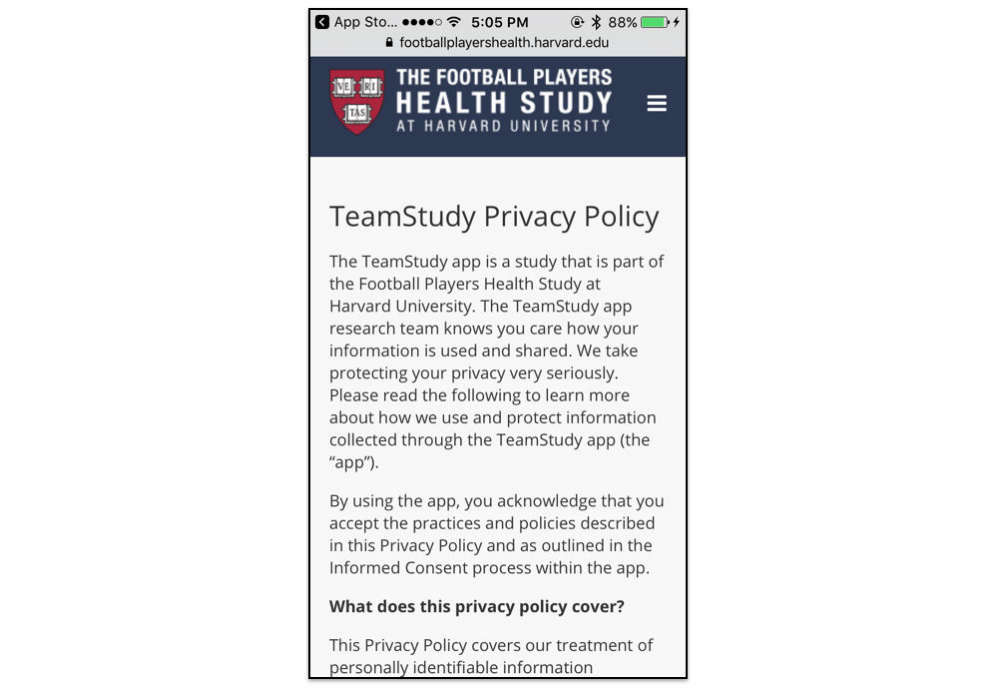
Privacy Policy, Team Study, Harvard University.

The multitude of potential mishandlings of data make a strong case for participants to be informed of these possibilities. Information for participants about the collection, transfer, and use of their data often resides in the app PP. Within its AppStore/iOS policies, Apple states that any app collecting any user or usage data, including personal health information must have a PP, however they provide no guidelines on what elements are required within that PP [16]. Unsurprisingly, we found PPs with a broad spectrum of detail and transparency from the very minimalist mTech, University of the Pacific (Figure 5) to the exhaustive Team Study, Harvard University (Figure 6) which is tailored specifically to the research app.

### Redistribution of Data

**Figure 7:**
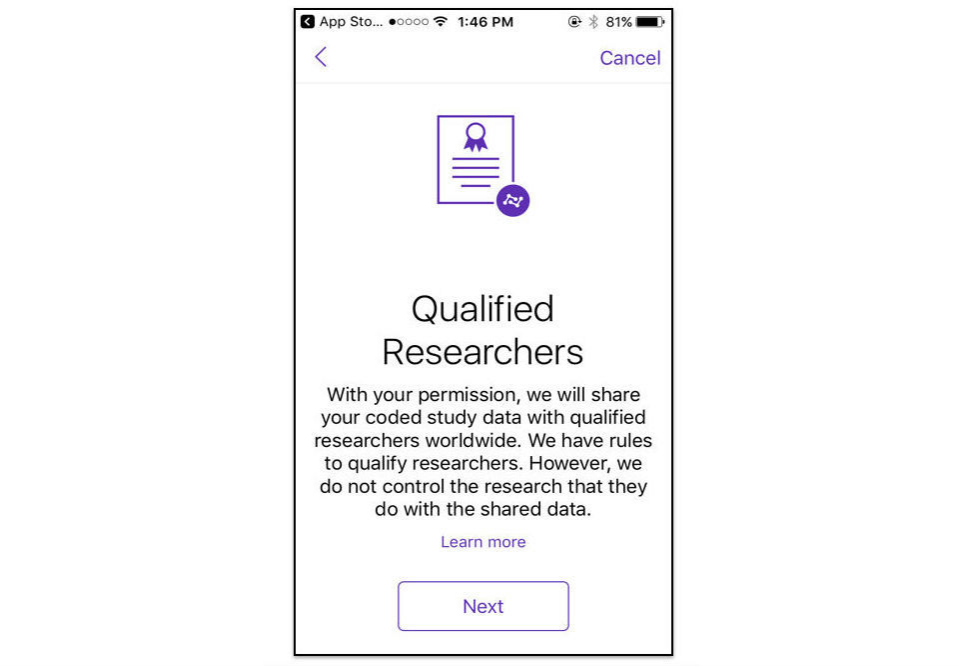
Participants have the option to share data broadly with other qualified researchers. Image from mPower, Sage Bionetworks.

Because of its highly structured, electronic nature, data collected through app-mediated research can be easily redistributed. There are 16 apps that specifically engage participants on the topic of data reuse for additional research as part of the eConsent. In the consent processes of these apps, participants designate if their data will be available in aggregate for reuse in future independent research. For those who advocate for open data sharing, app-mediated research has the potential to revolutionize how data is shared and analyzed by many researchers at one time, and thus maximize the scientific value of participant data donation (Figure 7). Of the apps included in this review, many use the nomenclature of “Qualified Researchers Worldwide” advocated by Wilbanks and Friend of Sage Bionetworks [17]. This nomenclature was first used by Sage Bionetworks as a process to qualify researchers to access open data from the ResearchKit app and mobile study mPower [18]. Even though the process and requirements to access such data may be different from the process used by Sage Bionetworks, this standard nomenclature sets a precedent for open data sharing in app-mediated research.

Data is easily redistributed for commercial use. Of the apps surveyed, 18 of 26 disclose to participants that they will not sell or share participant data for commercial purposes (See sheet “Confidentiality, Column K”). But perhaps more interesting to this review are the 8 apps that do not explicitly state that participant data will not be sold. As per the Apple developer guidelines, “Apps may not use or disclose to third parties data gathered in the health, fitness, and medical research context—including from the HealthKit API, Motion and Fitness, or health-related human subject research—for advertising or other use-based data mining purposes other than improving health management, or for the purpose of health research, and then only with permission” [16].

However, it is unclear whether these apps intend to disclose data to third parties, or simply fail to state that they will not disclose this data to participants.

### Integration of Genomic Data

Recently, researchers have expanded the scope of their app-mediated research to include genomic data from outside sources. Participants in the MyHeart Counts, Stanford and Asthma Health, Mount Sinai studies may elect to integrate their 23andMe genomic analysis into the app. However, at the time of this review, there was no update to any of the primary informed consent documentation to reflect this optional expansion of scope. Rather, a second consent specific to genomic information integration is contained within the app under the 23andMe module. PPD ACT, University of North Carolina (UNC) at Chapel Hill has elected to integrate genomic data through independent sequencing, in which a subset of participants will receive a “spit kit” in the mail to be returned to the researchers. This optional research activity is disclosed in Phase I of the consent and was included in the initial scope of the research. In Phase I consent, participants agree to answering survey questions contained within the app. Participants go through Phase II of the consent process if they have been chosen and elected to donate genomic data. The reviewers were unable to include analysis of the consent to donate genomic data, as it is only available to participants who have been selected by researchers to participate in Phase II of the study.

### Vulnerable Populations

**Figure 8:**
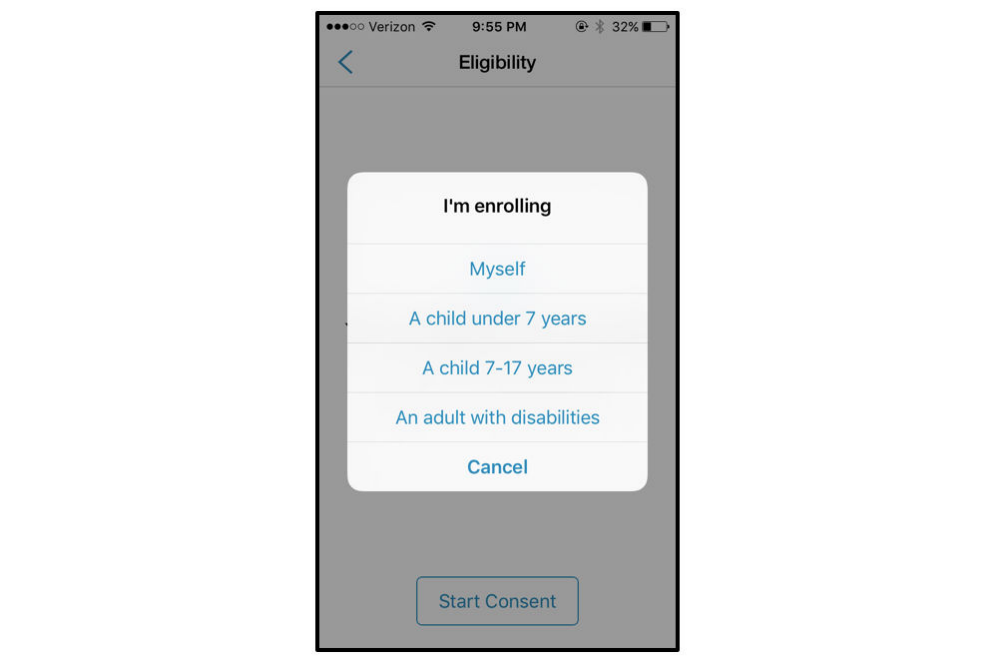
Enrolling children. Image from Autism and Beyond, Duke University.

App-mediated research may include vulnerable populations, most notably children (Autism and Beyond, Duke; FeverPrints, Boston Children’s Hospital) and pregnant women (Yale EPV, Yale). The consent for app-mediated research targeting vulnerable populations differ from those enrolling only populations considered not vulnerable. Both Autism and Beyond and FeverPrints modify the consent process to reflect the assent of children and consent by parents or guardians. FeverPrints requires that the person going through the consent process on the phone be 18 years of age or above. Then they are asked who they are enrolling (see Figure 8). However, beyond this designation, there is no difference in the eConsent or lexicon used to reflect differences in who is being enrolled. Traditionally, pregnant women have been considered vulnerable populations when they cannot expect benefit from participation in research or when the fetus might be adversely impacted by the mother’s participation [19]. In the case of the Yale EPV app, participants are expected to receive benefit (tracking of placental development) without any suspicion of risk to the fetus. In this way, pregnant women may not be deemed vulnerable within this research study.

### Recontact

About half (12/26, 46%) of apps surveyed address recontacting participants, primarily in the context of participating in future research studies (See sheet “Confidentiality,” Column N). Fourteen studies also address the return of the data the participant shares with the study to the participant (See sheet “Confidentiality,” Column O). Mobile research studies ease the return of data and potential results to participants and may facilitate a deeper reciprocal relationship between researchers and participants, although the risk of return of data and/or results are just beginning to be explored.

### Informedness in Remote Consent

According to the Nuremberg Code, a human subject consenting to research “should have sufficient knowledge and comprehension of the elements of the subject matter involved as to enable him to make an understanding and enlightened decision” [10]. Thus, it is an ethical requirement of human subjects research, and one of many IRBs, that researchers ensure participants are adequately informed about the research before participation.

Lexicon used within the eConsent slides and the LFC document to explain potentially complex concepts to participants is central to participants’ understanding of consent. Many have advocated for the use of simple language to promote participant comprehension and encourage informedness. Within clinical care, the National Quality Forum advocates for the use of universal symbols and pictures to improve comprehension in informed consent and specifies that written informed consent documents be at a US fifth-grade reading level (age 11) or lower [20]. Of the LFC documents reviewed, the average reading level is 10.9 (age 15-16) using the Flesh Kinkaid scale. Within the eConsent, another potential factor effecting comprehension was noted: a deviation in nomenclature from traditional consent documents, including the app’s own LFCs. “Risks” in the LFC document (risk to privacy, risk of general discomforts) are referred to as “Issues to Consider” in the eConsent.

As consent processes reviewed are administered entirely remotely, this creates a new challenge for researchers in assessing informedness of participants. In a traditional research setting, a study coordinator or other personnel would sit face-to-face with potential participants to administer consent, with the opportunity to assess informedness in real time. Clearly a different strategy is needed for remote, self-administered consent. Perhaps the most common response among the apps surveyed is the use of an assessment. Some apps use this assessment as a summative evaluation and as a measure of participant ability to give informed consent. With these evaluations, if a prospective research participant does not answer enough questions correctly, they are sent back to the beginning of the consent process. However, more commonly researchers used the assessment as a formative evaluation, a chance to enhance participant understanding by prompting incorrect answers with the correct information about the study. About one-third of apps surveyed (9/26, 35%) have implemented a summative evaluation at the end of the consenting process to test participants’ understanding of key concepts contained in the consent (See sheet “Unique to eConsent,” Column D). Most commonly, these quizzes test participant understanding of the purpose of the study, the fact that it is not medical care, that study data will be stored without direct identifiers, and participants’ ability to withdraw at any time.

### Conclusions

The consent processes presented in this review contain varying elements to contribute to participant informedness and transparency on the part of the research team. The new ecosystem of app-mediated research holds great promise to accelerate medical discovery through gathering potentially unprecedented amounts of highly structured data. However, app-mediated research also holds unique risks to participant data. The self-administered consent processes reviewed here present scalable approaches to informed consent to facilitate app-based research studies. The research community must continue to advocate for the importance of participant informedness, voluntariness, and comprehension in human subjects research. The variation, including strengths and gaps, observed in these informed consent processes have been highlighted to open a community dialogue about standards within this emerging field.

## Appendix

Multimedia Appendix 1 Consent toolkit inventory.

## Abbreviations

GA4GH: global alliance for genomics and health
IRB: institutional review board
LFC: long form consent
PP: privacy policy
UNC: University of North Carolina
